# THE EFFECTS OF HEALTH COACHING ON PATIENT ACTIVATION AND WELL-BEING OF COMMUNITY-DWELLING OLDER ADULTS

**DOI:** 10.1093/geroni/igae098.3741

**Published:** 2024-12-31

**Authors:** Edwin Ka Hung Chung, Dannii Y Yeung, Frank Youhua Chen

**Affiliations:** City University of Hong Kong, Hong Kong, China (People’s Republic); City University of Hong Kong, Hong Kong, China (People’s Republic); City University of Hong Kong, Hong Kong, China (People’s Republic)

## Abstract

Health coaching appears as a promising intervention to initiate behavioural change and improve health outcomes for outpatients (Krok-Schoen et al., 2017; Sharma et al., 2016). However, its effectiveness in improving self-management of chronic diseases were mainly examined in young and middle-aged adults with specific chronic diseases (Lin et al., 2021; Tülüce & Kutlutürkan, 2018). Whether health coaching is still effective in community-dwelling older adults, particular those with lower education and multiple chronic diseases, remains uncertain. This study thereby aims to examine the effects of a health coaching intervention in a sample of 194 older Hong Kong Chinese adults (Mage = 79.1, SD = 6.90, range = 62–95). Depending on their health needs, participants received 12 – 18-week home-based health coaching intervention during July 2022 – July 2024. Their patient activation, physical activity, nutrition behaviours, loneliness, and depression were measured before and immediately after the intervention. Repeated measures ANCOVAs showed that participation in the health coaching intervention significantly increased patient activation and nutrition-related health behaviours, and reduced loneliness and depression. Theses results remained significant even after controlling for age, sex, education, living arrangement, receipt of community centre and home care services, numbers of chronic diseases, polypharmacy, and frequency of intervention sessions. The findings of this study thus revealed beneficial effects of health coaching in enhancing older adults’ competence in managing their health, engagement in health behaviours, and psychological well-being. Policymakers and health-related professionals should implement health coaching in the primary healthcare system to maintain older adults’ well-being and promote ageing-in-place.

